# Supporting crop pollinators with floral resources: network-based phenological matching

**DOI:** 10.1002/ece3.703

**Published:** 2013-08-02

**Authors:** Laura Russo, Nelson DeBarros, Suann Yang, Katriona Shea, David Mortensen

**Affiliations:** 1 Biology Department, Pennsylvania State University415 Mueller Laboratory, University Park, Pennsylvania; 2 Connecticut Department of Energy and Environmental ProtectionHartford, Connecticut; 3 Biology Department, Presbyterian CollegeClinton, South Carolina; 4 Department of Plant Sciences, Pennsylvania State UniversityUniversity Park, Pennsylvania

**Keywords:** Crop pollination, ecosystem services, floral provisioning, mutualism, native pollinators, network theory

## Abstract

The production of diverse and affordable agricultural crop species depends on pollination services provided by bees. Indeed, the proportion of pollinator-dependent crops is increasing globally. Agriculture relies heavily on the domesticated honeybee; the services provided by this single species are under threat and becoming increasingly costly. Importantly, the free pollination services provided by diverse wild bee communities have been shown to be sufficient for high agricultural yields in some systems. However, stable, functional wild bee communities require floral resources, such as pollen and nectar, throughout their active season, not just when crop species are in flower. To target floral provisioning efforts to conserve and support native and managed bee species, we apply network theoretical methods incorporating plant and pollinator phenologies. Using a two-year dataset comprising interactions between bees (superfamily Apoidea, Anthophila) and 25 native perennial plant species in floral provisioning habitat, we identify plant and bee species that provide a key and central role to the stability of the structure of this community. We also examine three specific case studies: how provisioning habitat can provide temporally continuous support for honeybees (*Apis mellifera*) and bumblebees (*Bombus impatiens*), and how resource supplementation strategies might be designed for a single genus of important orchard pollinators (*Osmia*). This framework could be used to provide native bee communities with additional, well-targeted floral resources to ensure that they not only survive, but also thrive.

## Introduction

There are 87 globally important commercial crop species that depend on insect pollination (Klein et al. 2007). Among the insect pollinators, bees are the most important pollinating agents (Free 1993). Of the bees, honeybees (*Apis mellifera* L.) are the single most important crop pollinators, contributing not only to the diversity but also to the affordability of many agricultural food products (Losey and Vaughan 2006; Klein et al. 2007; Garibaldi et al. 2013). In the United States alone, the services of honeybees were valued at 14.6 billion US$ in 2000 (Morse and Calderone 2000) (19.3–40.3 billion US$ when adjusted for inflation in 2012), and demand for pollination services is increasing as ever larger areas are devoted to pollinator-dependent crops (Aizen et al. 2008).

However, honeybee populations are threatened by a suite of hazards, including pesticides, diseases, the mite *Varroa destructor* (vanEngelsdorp et al. 2009), and the potential indirect effects of loss of habitat (Potts et al. 2003; Winfree et al. 2007) and host plants resulting from herbicide drift (Mortensen et al. 2012). These threats have highlighted the dependency of modern agriculture on increasingly threatened pollination services (Gallai et al. 2009).

While honeybees are traditionally considered to be the most valuable pollinators (Free 1993), they are not the most efficient pollinators for all crops. Native bees are often efficient and sometimes superior pollinators, and contribute significantly to crop yield (Klein et al. 2003; Greenleaf and Kremen 2006; Losey and Vaughan 2006). For example, for “Red Delicious” apples, flowers visited by *Osmia cornuta* were five times more likely to set fruit than honeybee-visited flowers, and resulting fruits were larger when flowers were visited by *O. cornuta* (Vicens and Bosch 2000). The value of pollination services provided by wild bees has been estimated at approximately 3.07 billion US$ in the United States alone (Losey and Vaughan 2006) and they were globally valued at 248 billion US$ in 2009 (Gallai et al. 2009). Native bees are capable of fully supplying the pollination services required by certain crops (Winfree et al. 2007), but require sufficient habitat with floral and nesting resources to maintain a population size large enough to be effective crop pollinators (Kremen et al. 2004; Cane 2008). In response to habitat loss, for example, there have been declines in native bee populations in the northeastern United States (Bartomeus et al. 2013). Supplementing habitat for native bees may provide the additional benefit of supporting managed bee populations (Carvalheiro et al. 2012). For example, Carvalheiro et al. (2012) found increased honeybee contribution to the pollination of mangoes when floral provisioning resources were provided for native bees.

Both in response to threats to honeybees, and in recognition of the potential benefits of augmenting wild bees, methods are being developed to conserve native and domesticated bee populations. One strategy involves managing agricultural field edges to increase the diversity of floral provisioning resources (Winfree et al. 2008; Egan and Mortensen 2012) and the abundance of specific floral hosts (Isaacs et al. 2009). Current recommendations for selecting floral provisioning species are often based on pollinator syndromes, without incorporating information about actual insect visitation frequencies (e.g., NAPPC (North American Pollinator Protection Campaign) 2011). However, selecting the best plants for provisioning wild pollinators with nectar and pollen resources can be difficult because visitation rates often depend on multiple complex floral characters (Thompson 2001). The quality and quantity of resources provided by flower species can vary significantly, and quantifying these resources can be challenging (Kearns and Inouye 1993). For this study, we asked the question: given the threats to pollinators, how can we promote stability and diversity of bee communities that provide pollination services to crops?

To make a more informed decision about the plant species that might be used to conserve bee communities, and to identify bee species that might also visit a wide variety of crop species, it is necessary to observe interactions between bees and plants. These observations can then be used to capture community interaction structure in the form of bipartite mutualistic networks (Memmott 1999). Such networks have recently gained attention in the scientific literature as a vital tool for understanding how ecological communities form and function (Memmott 1999; Olesen et al. 2008; Campbell et al. 2011). Here we show that these methods can also be used to address the real-world management problem of how to improve floral provisioning with the objective of conserving native and managed bee populations to provide crop pollination services.

Specifically, we use network measures to assess the stability of community interaction structure over time and the role of individual species. These measures allow us to investigate the roles of individual plant species in the connectivity of the pollinator community, and we thus are able to rank them. Our analyses include one novel “node duration” measure to demonstrate how phenology relates to the importance of species, but we also show how interaction phenology can be used to match pollinators with a suite of plants that provide continuous floral provisioning resources throughout the season and how the phenology of the interacting species relates to the stability of the community as a whole over time.

We investigate three case studies to demonstrate how our framework might be used to target management objectives. First, we demonstrate how floral resources might benefit the domesticated honeybee (*A. mellifera*). We show that the provisioning habitat could be used to complement and supplement crop species, and to provide continuous resources throughout the active season. Second, we demonstrate how a generalist bee species, the common eastern bumblebee (*Bombus impatiens*), could be supported by multiple floral provisioning species throughout the summer. *A. mellifera* has long been used in agriculture, whereas *B. impatiens* has only been recently domesticated (i.e., in the 1970s, Velthuis and van Doorn 2006); both might benefit from additional and varied resources (Carvalheiro et al. 2012).

Next, we focus on the genus *Osmia;* some *Osmia* species have gained attention because of their potential to be managed as orchard pollinators (Vicens and Bosch 2000; Bosch and Kemp 2002; Gruber et al. 2011). For example, *Osmia lignaria, O. cornifrons,* and *O. cornuta* are sometimes managed as pollinators of almonds, cherries, plums, pears, and apples (Bosch and Kemp 2002). In most cases, the bloom period of these crop plants corresponds directly with the natural activity period of native *Osmia* spp. (Bosch and Kemp 2002). Although they prefer flowers of orchard trees when available, they require other resources when orchards are not flowering (Bosch and Kemp 2002). Floral resource provisioning in an orchard setting could help sustain *O. lignaria* populations when crop flowering is poor (e.g., when a hard freeze or pest infestation kills buds or blossoms) and also help to build local populations over time. As other *Osmia* spp. may also be effective crop pollinators, we have selected them as a target group to illustrate the application of our framework (Gruber et al. 2011).

## Material and Methods

### Experimental design

We established floral provisioning habitat 25 m from the edge of a 6-hectare corn field in the Russell E. Larson research farm, Centre County, PA (coordinates; 40.712019,−77.934192). The experiment consisted of 25 native perennial species (Table 1) and was established in 2007 in a randomized complete block design, with four blocks of the 25 species. We chose native plant species because they represent appropriate taxa for floral provisioning with native pollinators and require less maintenance than plants not adapted to the local climate (Isaacs et al. 2009). Each block consisted of individual plants, separated by 3 m, within a 12 m × 12 m grid. Blocks were aligned in a single row and positioned 6 m apart. The effect of blocks was not significant, and we subsequently pool visitors from each species and across both years that bees were collected (2008 and 2009). The purpose of the randomized complete block design was therefore to ensure that each plant was treated as an individual experimental unit, and also to ensure a relatively even spatial distribution of the species.

**Table 1:** List of twenty-five native perennial plant species used in this study, sorted by family

Species binomial	Common name
Asclepidaceae	
*Asclepias tuberosa* L.	Butterfly milkweed
Asteraceae	
*Conoclinium coelestinum* (L.) DC.	Blue mistflower
*Coreopsis tripteris* L.	Tall tickseed
*Echinacea purpurea* (L.) Moench	Eastern purple coneflower
*Eupatorium perfoliatum* L.	Common boneset
*Eupatorium purpureum* L.	Sweetscented joy pye weed
*Eurybia macrophylla* (L.) Cass.	Bigleaf aster
*Liatris pycnostachya* Michx.	Prairie blazing star
*Solidago rugosa* Mill.	Wrinkleleaf goldenrod
*Symphyotrichum novae-angliae* (L.) GL. Nesom	New England aster
*Symphyotrichum novi-belgii* (L.) GL. Nesom	New York aster
*Vernonia gigantea* (Walter) Trel.	Giant ironweed
Campanulaceae	
*Campanula rotundifolia* L.	Bluebell bellflower
Commelinaceae	
*Tradescantia ohiensis* Raf.	Ohio spiderwort
Fabaceae	
*Desmodium canadense* (L.) DC.	Showy ticktrefoil
*Lespedeza capitata* Michx.	Roundhead lespedeza
*Senna hebecarpa* (Fernald) Irwin and Barneby	American senna
Lamiaceae	
*Monarda fistulosa* L.	Wild bergamot
*Pycnanthemum tenuifolium* Schrad.	Narrowleaf mountainmint
Polemoniacae	
*Phlox divaricata* L.	Wild blue phlox
Primulaceae	
*Lysimachia quadrifolia* L.	Whorled yellow loosestrife
Ranunculaceae	
*Actaea racemosa* L.	Black bugbane
*Aquilegia canadensis* L.	Red columbine
Scrophulariaceae	
*Penstemon digitalis* Nutt. ex Sims	Talus slope penstemon
*Veronicastrum virginicum* (L.) Farw.	Culver's root

In the summers of 2008 and 2009, we vacuum sampled the flowers every other week from May to October with a modified leaf blower (Craftsman, model #358794760, Hoffman Estates, IL) (Tuell et al. 2008; DeBarros 2010). On a sampling day, each individual plant was vacuumed for 15 sec in a randomized sequence from 0900–1200 EST and again from 1300-1600 Eastern Standard Time (EST). Therefore, there were a total of 18.3 sampling hours on this community between the 2 years.

We also measured a number of other plant characteristics, such as the area of each individual flower and the total number of flowers across both seasons. The average planar floral area of an individual blossom was measured for each species by taking digital photographs of 10 representative blossoms or blossom clusters on each individual plant, keeping a metric ruler in each photographic frame for reference. We then used Adobe Photoshop CS4 Extended (Version 11.0, Adobe 2008) to calculate the area of the blossom in each photograph, and averaged across the 10 photographs for each individual. The total floral area for individual plants was then estimated for each week by multiplying the number of observed flowers by the average blossom area for the species (DeBarros 2010). Floral visitors of the superfamily Apoidea were pinned and identified to the species level, except for 62 *Lasioglossum* specimens that could only be resolved to morphospecies because they were too damaged to be identified or were males. Males of the genus *Lasioglossum* are not well resolved, and are often impossible to separate taxonomically (S. Droege, pers. comm.). Thus, they likely represent more than one species. Nonetheless, due to their impact on the visitation rates of plants, it was inappropriate to leave the specimens out of the analysis entirely. For all species, we deposited voucher specimens in the collections at the Pennsylvania Department of Agriculture in Harrisburg, PA.

### Network construction and analysis

**Figure 1:**
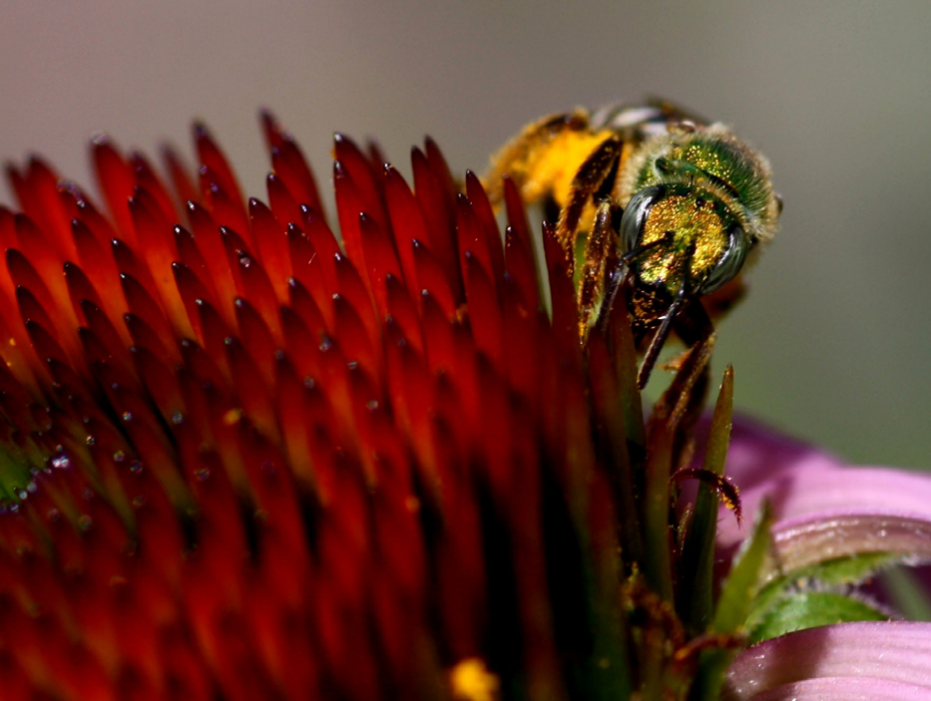
An example of an observed visitation event between a sweat bee (*Agapostemon virescens*) and one of the floral provisioning plant species (*Echinacea purpurea*). Photo by L. R.

From floral visitation events, we constructed both weighted and unweighted bipartite networks with plant and bee nodes, using a visitation event as an interaction (Fig. 1), with the “bipartite” package in R (Memmott 1999; Dormann et al. 2008). The interaction network comprised visitation events from both years and all blocks. A weighted version was scaled by the abundance of bees collected on flowers, whereas an unweighted version of the network documented the presence or absence of species interactions only.

To evaluate the effect of phenology on network structure, we separated the season into early (May and June), middle (July and the first half of August), and late (from the second half of August through the first half of October) summer. These periods were chosen based on the flowering phenology of the plant species in the floral provisioning habitat. For each of these periods of the summer, we evaluated the size, nestedness, and connectance of the network. The size of the network at any given time is the sum of the species richness of interacting plant and bee species. Nestedness is a measure of order in a network, and has been shown to relate to species and community persistence (Bascompte et al. 2003) and stability and robustness (Thébault and Fontaine 2010; Pocock et al. 2012). In addition, nestedness is not sensitive to network size (Nielsen and Bascompte 2007). Connectance has been theoretically shown to relate to the complexity and robustness of a community to species loss (Dunne et al. 2002) and stability (Thébault and Fontaine 2010). However, it is sensitive to small network sizes (Dormann et al. 2009).

On a node level (i.e., individual species), there are many ways to rank the species in the context of the community. Here, we evaluated the specialization, relative abundance, centrality, and duration of plant–pollinator interactions. As we were especially interested in identifying key plant species for floral provisioning, we also examined the number of visits each flower species received relative to their floral display, and ranked the plant species by their functional complementarity (Devoto et al. 2012). We tested for correlations between separate rankings using a Spearman's correlation coefficient.

We first identified generalist plants and pollinators as those species with the highest degree (i.e., largest number of species interactions; Memmott 1999); in network parlance, this can be referred to as degree centrality (Opsahl et al. 2010). Generalist plants support pollinator diversity, which in turn has been shown to provide increased crop yields (Klein et al. 2003; Garibaldi et al. 2013), especially given year to year variation in native bee populations. In turn, generalist pollinators are assumed to be more likely to visit not only plants in the floral provisioning habitat but also many crop species because they are less selective about where they obtain floral resources (Memmott 1999; Tylianakis et al. 2010).

We then identified plant species that supported large numbers of floral visitors by weighting interactions with interaction frequency to generate a quantitative network (Memmott 1999). The weighted degree of a node is the total abundance of all its interactions. Plant nodes with a high weighted degree are visited more. Highly visited plants likely support pollinator population growth and improve pollination services, especially if they provide both nectar and pollen, although they may also function as mating or nesting sites. In addition, some plants received a high frequency of visits despite having a relatively small floral display. We identified such species as those that were outside of a 95% confidence interval of the correlation between floral display and visitation frequency. In other words, they had more visitors than would be expected given the relationship between floral area and visitation frequency.

We used two additional separate measures of centrality in addition to degree centrality, both of which are common in the literature, for identifying the importance of individual nodes to network structure and stability (Jordán et al. 2007). Betweenness centrality ranks species as connectors between other species in the community, whereas closeness centrality ranks species relative to their topological proximity to other species in the community (Martín González et al. 2007). As the interactions between plants and pollinators generate bipartite networks, we made single-mode projections of the plant species and pollinator species before calculating centrality. In the single-mode projections, links are formed between species that share interacting partners (e.g., plant species that share one or more pollinator species).

We used duration of activity through the season of the plant and bee species as another measure of importance. For example, pollinators that actively forage for longer periods will likely visit multiple crop species with differing floral phenologies. Similarly, plant species that provide floral resources for longer periods can support pollinators when crops are not flowering. We define this new measure, which we term node duration, as the number of times out of the total number of samples that a species participates in the network. This measure is distinct from other measures of node dynamics in the literature (e.g., phenophase as defined by Olesen et al. 2008) because it only accounts for the presence of the species within the network. For example, the phenophase of a plant species is the period between the opening of its first flower and the senescence of its last flower (Olesen et al. 2008), whereas node duration is strictly defined by the number of times the plant species interacts with floral visitors in the community.

Finally, we calculated the functional complementarity of the plant species. Functional complementarity is a measure of how individual plant species support separate functional groups of pollinators in the community (Devoto et al. 2012). Plants that are visited by distinct groups of pollinators will therefore increase the functional complementarity of the community more than species that share the same species of pollinators. If there are constraints on the number of plants available for floral provisioning habitat, one might select a combination that maximizes functional complementarity to support the largest diversity of pollinators. As suggested by Devoto et al. (2012), we use branch lengths in a functional dendrogram based on a distance matrix generated from an interaction matrix. Here, we removed the species one by one in such a way as to maximize the functional complementarity of the community at each number of species. Thus, the order that the species are removed reflects a gradient from the species that are least critical to complementarity to those that are most critical.

### Example with target groups

To demonstrate how the floral provisioning species could complement and supplement flowering crop species, we created a separate phenology graph incorporating the interaction phenology of *A. mellifera* and the plant species it visited over the season. We compared the interaction phenology of these species with the approximate flowering phenology of five pollinator-dependent crop species commonly found in the study area. We performed a similar analysis with *B. impatiens* for comparison. (Readers interested in seeing the phenology-oriented visitation for any other of the 64 bee species found in this provisioning habitat can visit the following website for additional figures: http://www.floralprovisioningforpollinators.com.)

With the objective of conserving *Osmia* spp., we created a separate interaction network of the four *Osmia* spp. found in our provisioning habitat (*O. atriventris, O. bucephala, O. cornifrons*, and *O. pumila*) and the flowers they visited. We also evaluated the interaction phenology of the *Osmia* spp. to determine when they visited the provisioning habitat.

## Results

Over the two summers that the floral provisioning habitat was sampled, 64 bee species were captured while visiting the 25 perennial plant species. There were a total of 1651 specimens captured, representing a total of 261 unique insect–flower species interactions. We performed an interaction rarefaction to estimate the completeness of our sampling (a Chao 1 estimator, see Chacoff et al. 2012), and found that we captured 60.5% of the maximum number of expected interactions, a result consistent with other similar plant–pollinator communities, despite differing sampling methods (e.g., Chacoff et al. 2012; Devoto et al. 2012).

The community was dynamic across the summer, changing in size, nestedness, and connectance (Table 2). The community was largest in both the number of plant and insect species in the middle of the summer, and smallest early in the summer. The nestedness followed the same trend, being highest in the middle of the summer and lowest early in the summer, but the connectance followed an opposite trend, being lowest in the middle of the summer and highest early in the summer. When the interactions were pooled across the whole season, the nestedness was also maximized, but the connectance remained lower than the separate periods of the summer. However, the network size was below 50 species for the early division of the summer, and the measure of connectance for that time may therefore be less reliable (Dormann et al. 2009).

**Table 2:** Properties of the community over time

	Nestedness	Connectance	Number of plant species	Number of insect species	Total size
Early	2.80	0.23	8	23	31
Middle	13.05	0.17	20	46	66
Late	7.89	0.19	19	34	53
Full	20.77	0.16	25	64	89

**Figure 2:**
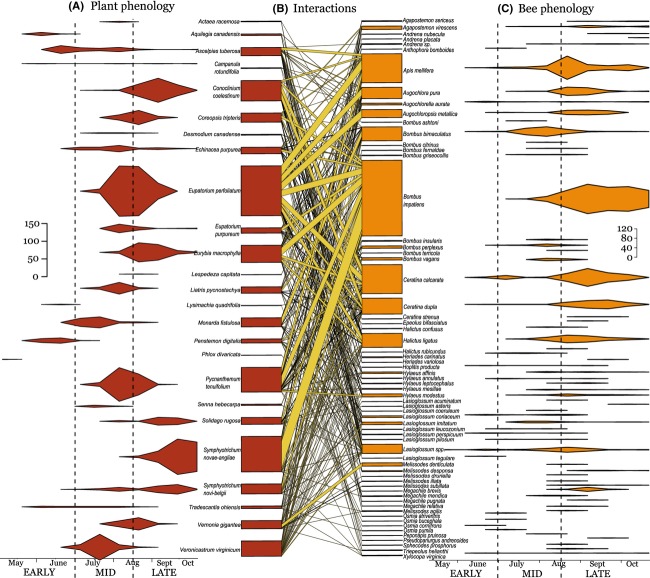
The violin-shaped plots represent the interaction phenology of plants (A) and bees (C) over the summers of 2008 and 2009. The weeks correspond to biweekly sampling dates, beginning in early May and ending in mid-October. The length of the segments demonstrates the duration of the interactive interval of that species, whereas the height represents the abundance of interactions and demonstrates how they fluctuate over time (plotted on the x-axes). (B) This quantitative bipartite visitation network was constructed from collections of Apoidea species on flower species. The boxes (nodes) on the left represent plant species and the boxes on the right represent bee species. The height of each box is proportional to the number of interactions. Lines connecting plant and bee species represent floral visitation events and are weighted by abundance.

We assessed the importance of plant and bee species in the context of the full network (Fig. 2) with five network measures: unweighted degree, weighted degree, betweenness, and closeness centrality, as well as node duration. Unweighted degree and weighted degree are correlated with each other and to both other measures of centrality, as well as functional complementarity in the plants (*P* << 0.01 for all except floral area and node duration, for which *P* ∼ 0.02, Table 1A). However, none of the node measures are significantly correlated with floral area or node duration in the plants (Table 1A). In contrast, all measures are significantly correlated with each other in the bee species (*P* << 0.01, Table 2A). Despite these significant correlations, each measure results in a substantially different ranking.

**Figure 3:**
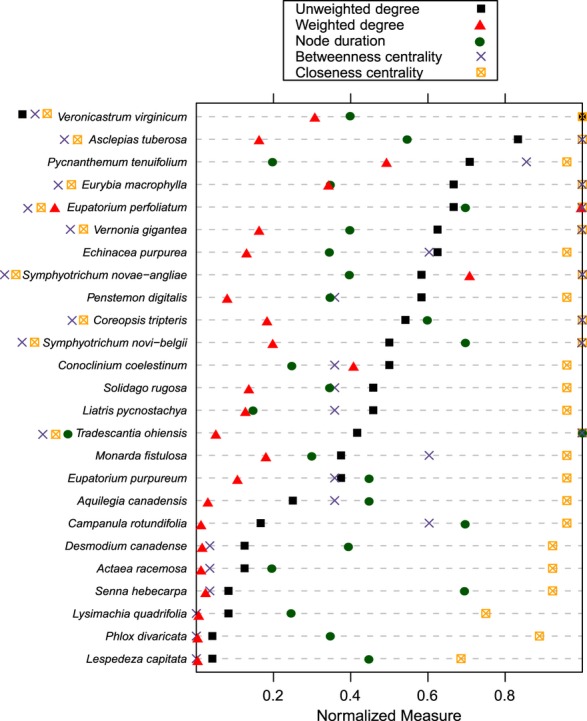
Plot of three normalized measures of importance for plants. Plant species are ranked by unweighted degree (filled square) (number of unique interactions with bees). Also shown are node duration (circle) (number of times bees were collected on the plant out of number of possible times), weighted degree (triangle) (interactions weighted by abundance of bee visitors), betweenness centrality (X), and closeness centrality (square with X). The symbols to the left of the species names indicate the highest ranking plant species for each measure.

**Figure 4:**
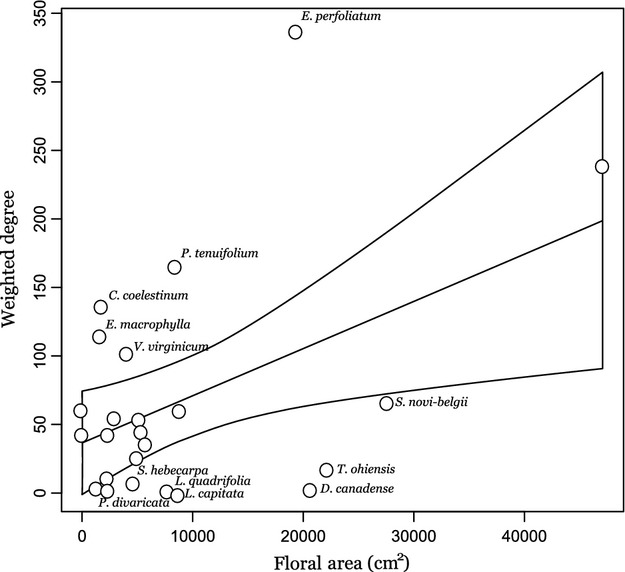
Plot of the relationship between weighted degree (visitation frequency) and floral area (summed across the summer), with 95% confidence intervals. Plant species that had more or fewer visits than would be expected given the relationship between floral area and weighted degree are labeled.

To demonstrate the relationships between these measures, we show the 25 plant species ranked by unweighted degree (Fig. 3, Table 3). Among the 25 species, *Veronicastrum virginicum* (culver's root) was visited by the greatest number of bee species, whereas *Eupatorium perfoliatum* (common boneset) had the highest abundance of bee visitors, and *Tradescantia ohiensis* (Ohio spiderwort) had the longest duration of activity, from May to September. Interestingly, the plant species were not well separated by either betweenness or closeness centrality. Indeed, nine species shared the top value for closeness centrality (Fig. 3). Five plant species had more visitors than would be expected given the relationship between floral area and weighted degree: *E. perfoliatum, Pycnanthemum tenuifolium* (narrowleaf mountainmint)*, Conoclinium coelestinum* (blue mistflower)*, Eurybia macrophylla* (bigleaf aster)*,* and *V. virginicum* (Fig. 4). In contrast, seven plant species had fewer visits than would be expected, given their floral area: *Symphyotrichum novi-belgii* (New York aster)*, T. ohiensis, Desmodium canadense* (showy ticktrefoil), *Lysimachia quadrifolia* (whorled yellow loosestrife), *Lespedeza capitata* (roundhead lespedeza), *Senna hebecarpa* (American senna), and *Phlox divaricata* (wild blue phlox).

**Table 3:** Properties of plants in the floral provisioning habitat

Species	Total floral area	Unweighted degree	Weighted degree	Node duration	Betweenness centrality	Closeness centrality	Functional complementarity
*Actaea racemosa*	1422	3	4	4	0.05	0.92	4
*Aquilegia canadensis*	2201	6	10	9	0.49	0.96	7
*Asclepias tuberosa*	2829	20	55	11	1.36	1	16
*Campanula rotundifolia*	1332	4	4	14	0.82	0.96	2
*Conoclinium coelestinum*	1697	12	138	5	0.49	0.96	21
*Coreopsis tripteris*	9073	13	62	12	1.36	1	10
*Desmodium canadense*	20,877	3	5	8	0.05	0.92	5
*Echinacea purpurea*	5519	15	44	7	0.82	0.96	11
*Eupatorium perfoliatum*	19,375	16	338	14	1.36	1	23
*Eupatorium purpureum*	5663	9	36	9	0.49	0.96	13
*Eurybia macrophylla*	1718	16	116	7	1.36	1	19
*Lespedeza capitata*	8733	1	1	9	0	0.69	6
*Liatris pycnostachya*	28	11	43	3	0.49	0.96	14
*Lysimachia quadrifolia*	7836	2	2	5	0	0.75	1
*Monarda fistulosa*	105	9	61	6	0.82	0.96	24
*Penstemon digitalis*	5114	14	27	7	0.49	0.96	12
*Phlox divaricata*	2113	1	1	7	0	0.89	3
*Pycnanthemum tenuifolium*	8510	17	167	4	1.17	0.96	22
*Senna hebecarpa*	4419	2	8	14	0.05	0.92	15
*Solidago rugosa*	2520	11	46	7	0.49	0.96	9
*Symphyotrichum novae-angliae*	47,075	14	240	8	1.36	1	24
*Symphyotrichum novi-belgii*	27,590	12	67	14	1.36	1	18
*Tradescantia ohiensis*	22,207	10	17	20	1.36	1	8
*Vernonia gigantea*	5076	15	55	8	1.36	1	20
*Veronicastrum virginicum*	3897	24	104	8	1.36	1	17

**Figure 5:**
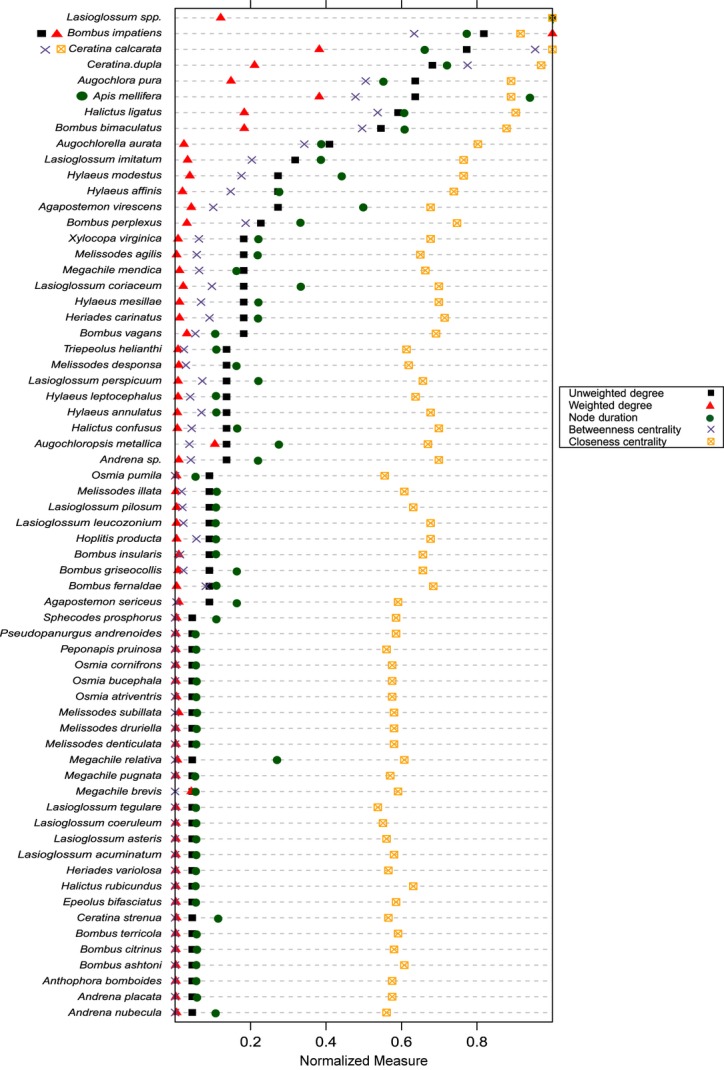
Plot of three normalized measures of importance for bees. Bee species are ranked by unweighted degree (filled square) (number of unique interactions with plants). Also shown are node duration (circle) (number of times bees were collected out of number of possible times), weighted degree (triangle) (interactions weighted by abundance of interactions with plants), betweenness centrality (X), and closeness centrality (square with X). The symbols to the left of the species names indicate the highest ranking bee species for each measure.

We also ranked the 64 bee species that visited the floral provisioning habitat by unweighted degree (Fig. 5). The unresolved *Lasioglossum* spp. have the largest of four of the five measures (unweighted degree, node duration, and both measures of centrality). As a group, they interact with the largest number of species, have the longest duration of activity, and the highest of both betweenness and closeness centrality. However, because they are unresolved, they likely represent more than one species. The interpretation is therefore that the genus *Lasioglossum* as a whole is an important group in the community, despite the fact that individual species tend to be specialists, or rare. This poorly known group may therefore require more study in the future, including the development of better guides for the identification of male specimens. If we remove the effect of the unresolved *Lasioglossum* spp., *B. impatiens* was both the most generalist species and most abundant. Its abundance was more than twice that of any other bee species. However, *Ceratina calcarata* had the highest ranking in both measures of centrality and *A. mellifera* had the longest duration of activity, from June to October. Importantly, though we found significant correlations between weighted degree, unweighted degree, betweenness, and closeness centrality, and node duration, (*P* << 0.01), each measure ranks the species differently.

Only three plant species, *Asclepias tuberosa* (butterfly milkweed)*, E. perfoliatum,* and *E. macrophylla,* always appeared among the top ten species when ranked separately by unweighted degree (number of unique interactions), weighted degree (including abundance), betweenness, and closeness centrality, and node duration (duration of interactive interval) (Table 3). Of these, *E. perfoliatum* and *E. macrophylla* still rank in the top ten, even when we controlled for floral display. In contrast, there were seven bee species (*B. impatiens, B. bimaculatus, Halictus ligatus, A. mellifera, Augochlora pura, Ceratina dupla,* and *C. calcarata*) that appeared important by all five measures (Fig. 5).

**Figure 6:**
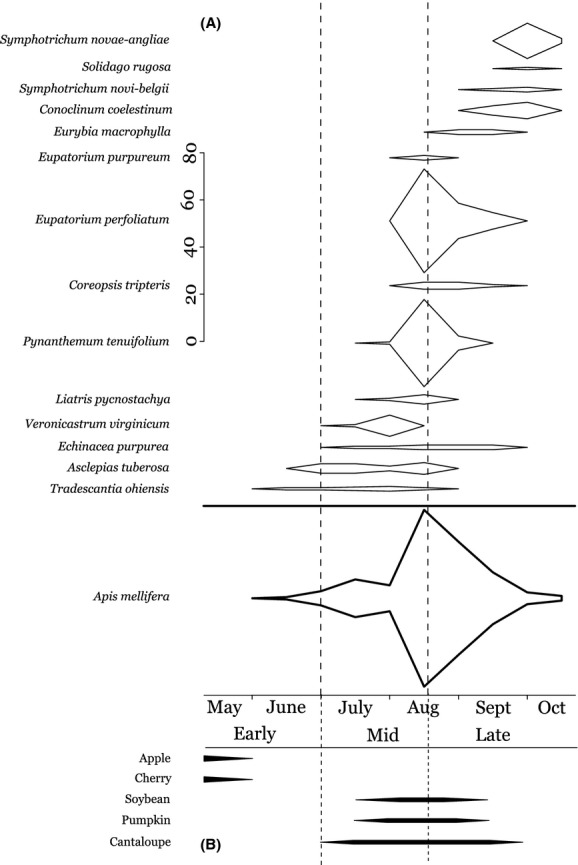
Interaction phenology of *Apis mellifera* (honeybee) across three periods of the summer. (A) Phenology of plant species in the provisioning habitat that interact with *A*. *mellifera*. To provide continuous resources across time, a manager might select one or more plant species from early, middle, and late in the summer. (B) Approximate flowering times of common pollinator-dependent crop species in the region of the study, including apple and cherry (B. Way of Way Fruit Farm, PA, pers. comm.), pumpkin (S. Sidhu, pers. comm.), cantaloupe (DeBarros 2010), and soybean (W.S. Harkcom, pers. comm.).

**Figure 7:**
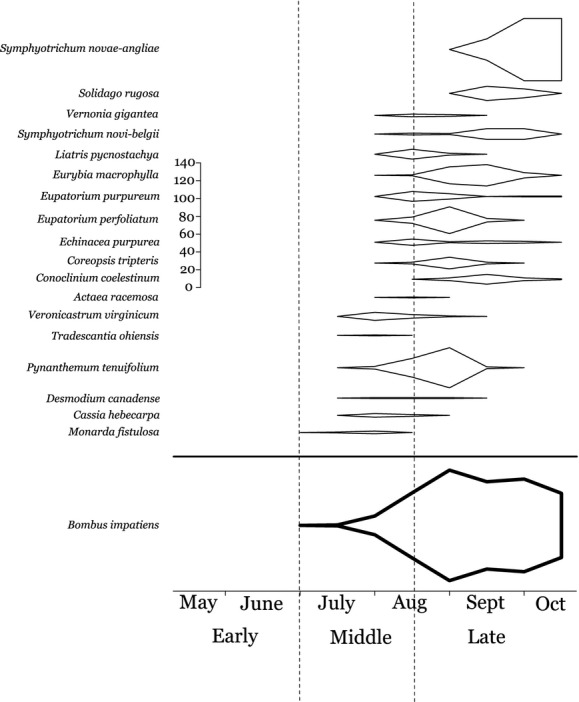
Interaction phenology of *Bombus impatiens* (bumblebee) across three periods of the summer, including phenology of plant species in the provisioning habitat that interact with *B*. *impatiens*. To provide continuous resources across time, a manager might select one or more plant species from middle and late in the summer.

The method of ranking may be adapted according to specific conservation goals. To show how one might select species to provide continuous floral resources for a target pollinator with a long duration of activity, we use the species visited by the honeybee (*A. mellifera*) as an example. The honeybee shifts its frequency of visitation from one species to another throughout the season; to ensure continuous resources, a manager would choose plants from each of the three periods (Fig. 6A). Floral resources could thus be available for this important domesticated pollinator when crop species are not in flower. In addition, we provide the approximate flowering times for five pollinator-dependent and high-yield crops in Pennsylvania (Fig. 6B). In contrast to the honeybee, the bumblebee (*B. impatiens*) was found to be extremely generalist, visiting multiple floral provisioning species within each time period (Fig. 7). This suggests that the bumblebee relies less on any single floral provisioning species and may be well supported by a wide variety of species.

**Figure 8:**
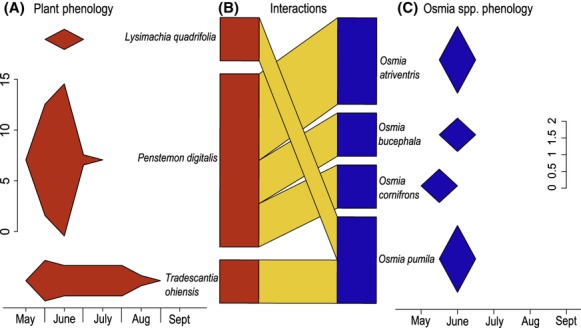
The interaction phenology (including all bee visits) of plants (A) that *Osmia* spp. visited, and of *Osmia* spp. (C) over the summers of 2008 and 2009. The weeks correspond to biweekly sampling dates, beginning in early May and ending in mid-October. The length of the segments demonstrates the interaction duration for that species, whereas the height represents the abundance of interactions and their fluctuations over time (plotted on the x-axes). (B) This quantitative bipartite visitation network was constructed from collections of *Osmia* spp. on flower species. The boxes (nodes) on the left represent plant species and the boxes on the right represent *Osmia* spp. The height of the boxes is the proportional number of interactions. Lines connecting plant and bee species represent floral visitation events and are scaled by abundance.

Although *Osmia* spp. were not frequent visitors in our floral resource provisioning habitat, we provide preliminary analyses as to the species that they visited. Our network demonstrates that the four *Osmia* spp. in the floral provisioning site visited only three plant species, and three of them visited only *Penstemon digitalis* (foxglove penstemon) (Fig. 8), though this plant was a minor node of the full network (Fig. 2). In our provisioning habitat, there were few plants in flower at the time that the *Osmia* spp. were active, and because the visitation frequency from these species was low, we do not suggest that *P. digitalis* is the ideal resource for all *Osmia* spp.; empirical confirmation would be necessary. However, the example demonstrates how our framework might be used to identify such a resource for a particular objective.

## Discussion

The benefits of a diverse bee community for agricultural yields have been convincingly demonstrated (e.g., Garibaldi et al. 2013). However, in order to have a stable, functional wild bee community, it is necessary for there to be sufficient habitat (Winfree et al. 2007) to provide floral resources, such as pollen and nectar (Isaacs et al. 2009). We show how network theoretical methods incorporate plant and pollinator phenologies to target floral provisioning efforts to conserve and support native and managed bee species. Although many have suggested that networks can be used to direct conservation and management objectives (e.g., Tylianakis et al. 2010), our study demonstrates how network theory might have a practical application to a real and urgent problem. Understanding the temporal dynamics of community level interactions in a plant–pollinator network is critical for maximizing the provisioning of floral resources for crop pollinators and targeting conservation efforts at species that provide pollination services. In particular, knowledge of phenological constraints on plant–pollinator interactions, and plant species used by bee visitors, will be essential to managers that have bee conservation as an objective.

Another advantage of our method is that it allows us to design the floral provisioning habitat with local bee assemblages in mind. This multispecies framework emphasizes the importance of key interactions. Stable, natural communities have particular structural aspects that we want to design or maintain to conserve diversity and functionality. In other words, we can select highly generalist species that are abundant and active over long periods of time, while sustaining rare interactions between uncommon or specialist species. In addition, by keeping the phenology of the whole community in mind, we can see where it might be more vulnerable, or less stable. For example, the nestedness of this community was lowest early in the summer, and highest in the middle of the season. That might pinpoint an opportunity to strengthen the bee community by providing more flower species with an early phenology in provisioning habitat. In contrast, the connectance of the community was highest early in the spring, though this was potentially influenced by network size.

To explore the phenology of individual species, we developed a novel network measure, node duration, or the activity of interacting species over time. Node duration can provide useful information for managers concerned about relative flowering times of floral provisioning plants and crops. Some studies have shown a “magnet species” effect where pollinator visitation rates to nearby species are enhanced by the presence of a species with large floral rewards (Molina-Montenegro et al. 2008). In this case, it would be advantageous to synchronize the flowering of crop and nearby attractive wild plants. However, there is also some evidence for the opposite effect, where plants compete for pollinators (Feinsinger 1987). In this situation, it would be ideal to have plant species that support pollinators when crop species are not in flower. Interaction phenology allows species in the community to be selected on the basis of the duration or seasonality of their activity (Fig. 2), as relevant to the focal management situation; such insights will help with the design and tractability of field trials or provisioning applications. Toward this end, we explore the case study of the interaction phenology of *A. mellifera* relative to the phenology of a selection of pollinator-dependent crop species (Fig. 6); to provide continuous resources for this bee species, a manager might select one or more provisioning species flowering in each of the three periods of the summer. In contrast, the bumblebee (*B. impatiens*) visited several species in each period of the summer, and may therefore be less reliant on any one plant species (Fig. 7).

We identified generalist plant species, such as *V. virginicum*, that attracted a large number of bee species, but also *E. perfoliatum* that attracted a large abundance of bees, and *T. ohiensis* that provided attractive resources over a long period of time. This demonstrates that different plant species might be used for different provisioning objectives. In addition, three species (*A. tuberosa, E. perfoliatum,* and *E. macrophylla*) among the plants of our floral provisioning habitat were consistently ranked highly in all categories measured, and *E. perfoliatum* and *E. macrophylla* still rank in the top 10 species even when we control for the size of the floral display. These species might be the strongest candidates for resource provisioning; their efficacy as resources should then be the target of field trials. In contrast, seven species had fewer visitors than would be expected given their floral display. This demonstrates that the more showy flowers are not necessarily the most preferred by bee visitors, especially if more attractive flowers are present (but see Tuell et al. 2008). Interestingly, measures of node betweenness and closeness centrality were not effective for separating plant species, likely because a subset of the plants species all had a large number of connections and were thus equally central to the interaction structure (Fig. 3).

Although we focused on specific plant species that would be ideal for provisioning crop pollinators, the complex structure of this network demonstrates how a diversity of floral resources contributes to a diverse pollinator community. Given the asymmetric nature of our community (and mutualistic communities in general), generalist plant species such as *V. virginicum* are visited by a large number of specialist bee species. In turn, bee diversity has been shown to result in improved crop pollination (Klein et al. 2003; Garibaldi et al. 2013). Thus, there is a strong relationship between diversity and functionality. Diverse communities provide more ecosystem services, and communities providing ecosystem services (i.e., pollination or floral resource provisioning) support a higher level of diversity (Kremen et al. 2004; Isaacs et al. 2009).

Our results also highlight pollinator species that might augment crop pollination. There were seven consistently generalist and abundant species that were active for most of the summer, and central to the interaction structure. Their periods of activity could overlap with multiple different crop species. Among these, *B. impatiens* stood out as more than twice as abundant and was also a very generalist bee species. It is possible that choosing plants to support *B. impatiens* might encourage its population growth. However, a different species, *C. calcarata*, was the most central when ranked by both betweenness and closeness centrality (Fig. 5), suggesting that it has a central role in the web of pollination services provided by these bee species and may, in fact, contribute to the stability of the community (Jordán et al. 2007).

Our approach can also be used to address specific management objectives, such as to maximize provision of resources that help conserve a target group of pollinators. The results of our second illustrative case study show that three of the four *Osmia* spp. active in the floral provisioning habitat visited one plant species in particular, *P. digitalis*. As few of the plants in our study were flowering early in the season, future studies would benefit from including additional, early blooming species. Indeed, despite the low sample size, we have included this analysis because managers are searching for new ways to support *Osmia* species in orchard systems, and experimentally testing multiple floral provisioning species (D. Biddinger, pers. comm.). Our study suggests that an empirical test comparing scenarios with and without *P. digitalis* would help to determine whether it is a key component of floral provisioning habitat designed to support orchard crops that might benefit from visits by *Osmia* spp. *P. digitalis* may serve to increase the population size of *Osmia* spp., thereby enhancing crop pollination in future years. Our method also makes explicit how the flowering phenology of plants within the provisioning habitat synchronizes with crop phenologies. For example, *P. digitalis* flowered after orchard crops and would therefore not compete with them for pollinators; instead, our work suggests that *P. digitalis* plantings adjacent to orchards might support *Osmia* spp. at a critical time when floral resources provided by crops are absent.

## Conclusions

Pollination services are critical for the production of foods necessary for a healthy human diet (Eilers et al. 2011). The demand for pollinator-dependent crops is increasing much more rapidly than the availability of pollination services provided by honeybees (Aizen et al. 2008), especially in the face of Colony Collapse Disorder. It is therefore imperative that these pollination requirements be supplemented with services provided by wild bees. However, wild bees require nesting and floral resources (Winfree et al. 2007), and relatively little is known about these requirements. Providing native bee communities with additional, well-targeted floral resources could ensure that they not only survive but also thrive; the benefits of such habitat may also support honeybees, which utilize similar floral resources, as evidenced by the visitation of honeybees to the species in our floral provisioning site. Our approach to assessing floral resources for crop pollinators integrates critical information about the community structure and phenology of relevant plant and bee species. As we illustrate, our framework can be used to inform hypotheses and design experiments, and has great flexibility for objectives intended to conserve wild bee populations and maintain the critical ecosystem service of pollination.

## Appendix

**Table A1:** A matrix of correlation coefficients between different node level measures for the plants. Significant (*P* << 0.01 except floral area and node duration, for which *P* ∼ 0.02) correlations are highlighted in gray. (Correlations are symmetric.)

	Betweenness centrality	Closeness centrality	Unweighted degree	Weighted degree	Node duration	Floral area
Closeness centrality	0.72					
Unweighted degree	0.82	0.67				
Weighted degree	0.57	0.41	0.58			
Node duration	0.38	0.25	−0.01	0.02		
Floral area	0.35	0.15	0.10	0.46	0.37	
Functional complementarity	0.63	0.54	0.68	0.74	−0.06	0.25

**Table A2:** A matrix of correlation coefficients between different node level measures for the insects. Significant (*P* << 0.01) correlations are highlighted in gray. (Correlations are symmetric.)

	Betweenness centrality	Closeness centrality	Unweighted degree	Weighted degree
Closeness centrality	0.94			
Unweighted degree	0.97	0.95		
Weighted degree	0.67	0.64	0.72	
Node duration	0.91	0.92	0.95	0.68
